# *Pseudomonas* DING proteins as human transcriptional regulators and HIV-1 antagonists

**DOI:** 10.1186/1743-422X-10-234

**Published:** 2013-07-15

**Authors:** Andrew Suh, Valentin Le Douce, Olivier Rohr, Christian Schwartz, Ken Scott

**Affiliations:** 1 School of Biological Sciences, University of Auckland, Private Bag, Auckland, 92019, New Zealand; 2 Institut de Parasitologie et Pathologie Tropicale, EA 7292, Université de Strasbourg, Strasbourg, 67000, France; 3 IUT Louis Pasteur, 1 allée d'Athènes, Schiltigheim, 67300, France

**Keywords:** *Pseudomonas aeruginosa*, HIV-1, DING protein, PA14, Transcription, NFκB, Viral replication

## Abstract

**Background:**

Anti-HIV-1 therapy depends upon multiple agents that target different phases of the viral replication cycle. Recent reports indicate that plant and human DING proteins are unique in targeting viral gene transcription as the basis of their anti-HIV-1 therapy.

**Methods:**

Two cloned DING genes from *Pseudomonas* were transiently expressed in human cells, and effects on NFκB-mediated transcription, HIV-1 transcription, and HIV-1 production were measured.

**Results:**

Both DING proteins elevated NFκB-mediated transcription. In microglial cells, one protein, from *P. aeruginosa* PA14, suppressed HIV-1 transcription; the other protein, from *P. fluorescens* SBW25, was inactive. The PA14DING protein also reduces HIV-1 production in microglial cells.

**Conclusions:**

Structural differences between the two DING proteins highlight regions of the PA14DING protein essential to the anti-HIV-1 activity, and may guide the design of therapeutic agents.

## Background

DING proteins are characterised by a highly identical DINGGG- amino acid sequence and are typically 30 – 40 kDa [1-4]. The first DING protein was found in the synovial fluid of rheumatoid arthritis patients [5] and since then, they have been identified in a range of different organisms in a variety of contexts [3]. There is increasing evidence that DING proteins have an ability to influence human transcription and recently an ability to block HIV-1 transcription and therefore viral replication (For review see [3]).

DING proteins have been found to influence transcription in a number of different situations. The synovial stimulatory protein, secreted by rheumatoid arthritis synovial fibroblasts, induces an inflammatory response in synovial T-cells and also has a mitogenic effect on synovial fibroblasts [5-7]. Although this does not directly suggest DING proteins have an influence on human transcription, these phenotypes are characteristic of an increase in NFκB activity.

In cancer cachexia, proteolysis-inducing factor (PIF), a 24 kDa sulfated glycoprotein, induces the breakdown of skeletal muscle leading to the loss of lean body mass in cancer patients [8-10]. PIF binds to a membrane receptor, a DING protein, and activates NFκB leading to the up-regulation of pro-inflammatory cytokines IL-6, IL-8 and TNF-α and also the induction of the ubiquitin-proteasome pathway [10,11].

Some DING proteins have been found to inhibit human and viral transcription factors and exhibit powerful repression of HIV-1 transcription and replication. The first DING protein that was discovered to have anti-HIV activity was isolated from the callus culture of St. John’s Wort [12]. An initially obtained truncated clone, p27^SJ^, inhibited HIV-1 transcription and replication in monocyte-derived cells by inhibiting the human transcription factor C/EBPβ and the viral trans-activator protein, Tat. There is evidence of a direct interaction of p27^SJ^ with the two transcription factors resulting in their functional inhibition [12,13]. A cDNA encoding a full-length P38SJ protein was subsequently cloned and found to have an even more powerful anti-HIV activity [14]. Other DING proteins have also been found to have anti-HIV activity. X-DING-CD4 (otherwise known as HIV resistant factor (HRF)) was isolated from the conditioned medium of an HIV-1 resistant clone of T-lymphocytes [15,16]. This protein is secreted by the HIV-1 resistant cells and the addition of purified HRF to the culture medium of non-resistant CD4+ T-cells makes these cells also resistant to HIV [16]. The exact mechanism of its action is unclear but there is evidence that it enters the cell and inhibits the p50 subunit of NFκB, preventing its binding to DNA leading to the inhibition of HIV-1 production [15]. It has recently been found that individuals who have innate immunity to HIV infection have a high level of DING mRNA expression [17]. This provides support that the anti-HIV property of DING proteins is effective *in vivo.*

The human phosphate binding protein (HPBP) isolated from the plasma of human blood has recently been found to have anti-HIV activity. Like X-DING-CD4, HPBP can be added to the culture medium of primary T-lymphocytes and primary macrophages resulting in the inhibition of HIV-1 replication [18].

Despite the isolation of a number of DING proteins that inhibit HIV-1 replication, their lack of known gene sequences has been a hindrance to research. The full amino acid sequence of HPBP has been identified from the crystal structure and mass spectrometry [19] but attempts to produce the recombinant protein through the use of synthetic genes have been unsuccessful due to insolubility.

In bacteria, the number of *Pseudomonas* DING genes, of which products are secreted phosphate binding proteins [20], has been gradually increasing in genome databases as systematic sequencing of genomes progresses. Unlike HPBP, recombinant *Pseudomonas* DING proteins can be readily purified and share high sequence and structural identity with eukaryotic DING proteins [21-23]. Furthermore, since DING proteins from *Pseudomonas* are highly identical to those found in eukaryotes, we hypothesized that they could be used as models to elucidate the function of eukaryotic DING proteins as transcriptional regulators and explore their anti-HIV effects. By using *Pseudomonas* DING proteins, we also aimed to address whether they could in fact influence human transcription, which transcription pathways they influenced and whether they could inhibit HIV-1 transcription and replication.

The current treatment for HIV-1 involving combination anti-retroviral therapy (cART) includes drugs that inhibit viral entry, reverse transcription of the viral genome, integration into the host genome and the maturation of the viral proteins. It is effective in reducing viral load but the complete eradication of the virus and the threat of resistance mutations continues to be a challenge [24-26]. It is therefore essential to continue the development of novel anti-HIV molecules that target other steps of the viral life cycle.

## Results

### Bacterial DING proteins elevate NFκB-mediated transcription

**Figure 1:**
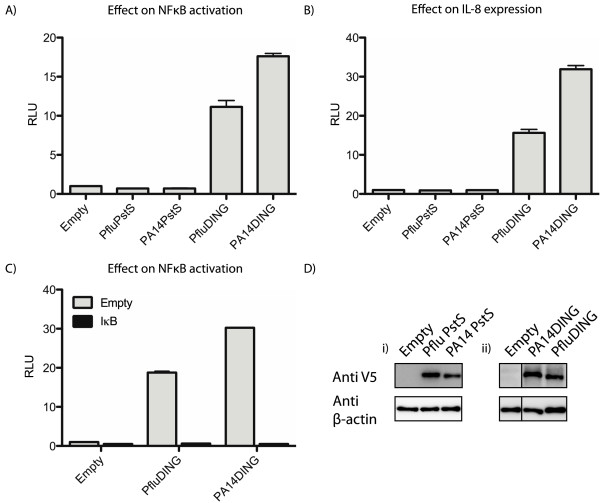
**DING proteins elevate NFκB mediated transcription.** Plasmids expressing DING and PstS proteins were transfected into HEK 293T cells along with a reporter plasmid containing: **A**) luciferase downstream of a promoter containing two NFκB binding sites (pBIIXLuc); **B**) a IL-8 reporter containing the IL-8 gene promoter; **C**) pBIIXLuc with and without the IκB super-repressor expressing plasmid. The data were normalised with a third plasmid containing *Renilla* luciferase under the control of a thymidine kinase promoter. The graphs are representative of at least 5 experiments with each protein*.* RLU: relative light units. **D**) Western blots showing the expression of V5-tagged (i) PstS, (ii) DING proteins and β-actin controls in transfected HEK 293T cells.

Previous studies indicate that DING proteins can affect NFκB-mediated transcription [5,10,14]. In order to ensure the intracellular localisation *of Pseudomonas* DING proteins and test their effect on NFκB transcription in a cell-based reporter assay, DING genes from *P. aeruginosa* PA14 (PA14DING) and *P. fluorescens* SBW25 (PfluDING) were cloned into human expression plasmids. The DING expression constructs were co-transfected with an NFκB limited-promoter reporter construct, pBIIXLuc and a renilla luciferase internal control in HEK 293T cells. Cell lysates were assayed for luciferase activities 48 hours post-transfection. Both PA14DING and PfluDING significantly stimulated NFκB activity with PA14DING having the highest effect (Figure 1A).

In order to control for the over-expression of a *Pseudomonas* phosphate binding protein, PstS genes from *P. aeruginosa* PA14 and *P. fluorescens* SBW25 were cloned into a human expression vector and tested for their effect on NFκB mediated transcription. DING proteins share a high structural identity with PstS proteins, which are involved in acquiring phosphate when the environmental phosphate level is low, and both proteins bind a single inorganic phosphate molecule. Whilst DING and PstS proteins are structurally and functionally similar, their sequence identity is under 25%. The PstS expression constructs were co-transfected with an NFκB limited-promoter reporter construct, pBIIXLuc and a renilla luciferase internal control. The cell lysates were assayed for luciferase activities 48 hours post-transfection. The PstS proteins did not significantly increase NFκB-mediated transcription providing support that the stimulatory effects of DING proteins were specific and not due to experimental artefacts (Figure 1A).

### Downstream effects of DING proteins

Since the DING-induced activation of NFκB was only tested in a limited NFκB reporter construct, the effect of DING proteins on a downstream component of the NFκB pathway was investigated to determine whether the DING-induced activation of NFκB had expected downstream consequences. HEK 293T cells were co-transfected with the PA14DING and PfluDING expression constructs, an IL-8 reporter construct and a renilla luciferase internal control. The luciferase activities were assayed 48 hours post-transfection. The DING proteins had a stimulatory effect on IL-8 expression (13-fold and 28-fold higher than for the empty vector control, for PfluDING and PA14DING, respectively) providing evidence that the DING-induced NFκB activation had downstream consequences (Figure 1B). Control experiments with PstS expression constructs, in place of DING, showed no significant elevation of the IL8 luciferase reporter.

In order to validate the specificity of the DING-induced NFκB activation, *Pseudomonas* DING expression vectors were co-transfected with the IκB super-repressor construct, in which the phosphorylation sites were mutated preventing its detachment from NFκB and subsequent degradation. The IκB super-repressor completely abolished DING-induced NFκB activation (Figure 1C).

### Phosphate binding is not required for NF-κB activation

**Figure 2:**
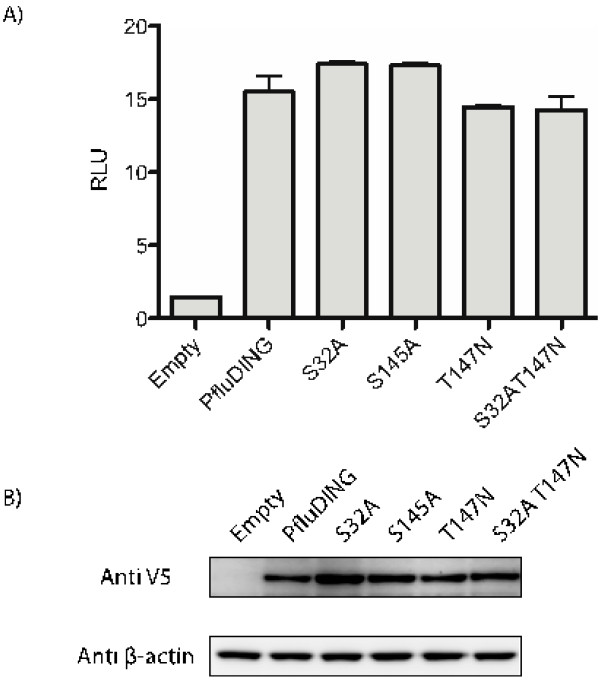
**The effect of PfluDING and site-directed mutant proteins on NFκB activity. A**) HEK 293T cells were transfected with pBIIXLuc (300~ng), renilla luciferase (80~ng) and PfluDING or mutant expression constructs (420~ng). The cell lysates were assayed for luciferase activities 48 hours post-transfection. The phosphate-binding mutants did not have significantly different effects on NFκB activity compared to the unmodified PfluDING. Each point was done in triplicate and this graph is representative of two separate experiments. The error bars represent standard error. RLU: relative light units. **B**) Western blot showing the expression of V5-tagged PfluDING, mutants and β-actin control in transfected HEK 293T cells.

We next investigated the level of effect that the phosphate-binding function of DING proteins had on NFκB activation. Mutagenesis studies were performed using PfluDING as a model. It has previously been shown that the mutagenesis of just one of the eight phosphate-binding residues of a recombinant *Pseudomonas* DING protein (T147N mutation) decreases the affinity of phosphate binding of recombinant PfluDING by 95% [21]. Two additional phosphate-binding residues of PfluDING, identified from its crystal structure, were selected and replaced with residues (S32A and S145A) that are unable to bind phosphate by site-directed mutagenesis in order to minimise phosphate binding. A double S32A/T147N mutation was also created to further eliminate phosphate binding. The unaltered PfluDING expression vector and mutant constructs were transfected into HEK 293T cells along with a NFκB reporter construct (pBIIXLuc) and the renilla luciferase internal control. The cell lysates were assayed for luciferase activities 48 hours post-transfection. The NFκB-stimulating levels of the phosphate-binding mutants of PfluDING did not significantly differ from that of the unmodified protein (Figure 2A).

### The PA14DING protein represses HIV-1 transcription and HIV-1 production

**Figure 3:**
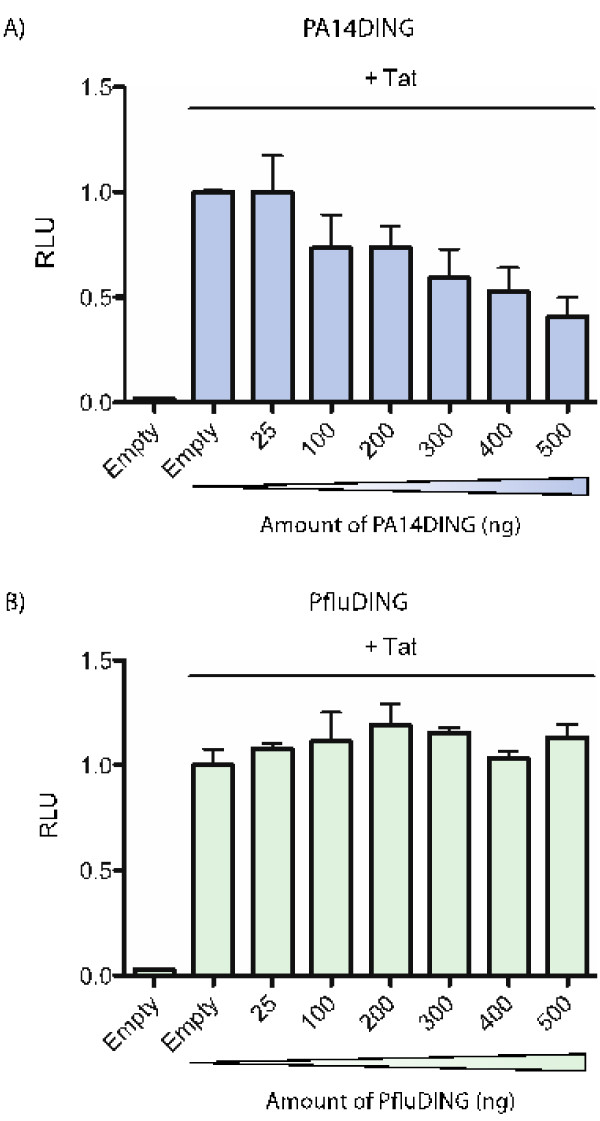
**Dose response of PfluDING and PA14DING on Tat-mediated HIV-1 transcription in microglial cells.** Microglial cells were transfected with HIV-1 LTR-luc (280 ng), renilla luciferase (100 ng), Tat (13 ng) and increasing amounts of the PfluDING or PA14DING expression vectors (0–500 ng). The total amount of transfected DNA was kept constant at 1 μg by the addition of the empty pcDNA3.2/V5-DEST vector. The cell lysates were assayed for luciferase activities 48 hours post-transfection. **A**) PA14DING inhibited Tat-mediated HIV-1 transcription in a dose-dependent manner. **B**) PfluDING had no effect regardless of the dose. The graphs are representative of at least three separate experiments. Error bars represent standard error. RLU: relative light units.

The effect of *Pseudomonas* DING proteins on HIV-1 transcription was assessed in microglial cells which had physiological relevance to HIV-1 infection. Microglial cells were co-transfected with the HIV-1 LTR-luc reporter vector, Tat, renilla luciferase and increasing amounts of the PA14DING and PfluDING plasmids. The cell lysates were assayed for luciferase activities 48 hours post-transcription. Increasing amounts of the PA14DING expression vector correlated with the repression of HIV-1 transcription (Figure 3A). PfluDING did not have an effect regardless of the dose (Figure 3B).

**Figure 4:**
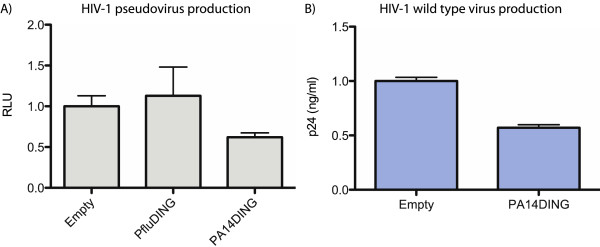
**The effect of DING proteins on HIV-1 production in microglial cells. A**) Microglial cells were transfected with the pNL43 HIV-1 ∆env pseudovirus genome (30 ng) and DING expression vectors (935 ng). The cell lysates were assayed for luciferase activity 48 hours post-transfection. PA14DING reduced HIV-1 replication by ~40% (p < 0.05). PfluDING did not have a significant effect. This graph is representative of at least three separate experiments. Error bars represent standard error. RLU: relative light units. **B**) Microglial cells were transfected with the full genome of wildtype HIV-1 strain pNL43 (30 ng) and PA14DING (970 ng). Viral production was determined by measuring levels of the viral capsid protein p24 in the conditioned medium by ELISA 48 hours post-transfection. Serial dilutions of recombinant p24 were used to plot a standard curve which was used to determine the absolute amount of p24 in the conditioned media. PA14DING inhibited HIV-1 replication by ~43% (p < 0.05). The graph is representative of two separate experiments. Error bars represent standard error.

The *Pseudomonas* DING proteins were tested for their effects on viral production. Initial experiments were done using a pNL43 Δenv pseudovirus which had the viral envelope genes replaced with a luciferase reporter gene. Microglial cells were transfected with the pNL43 Δenv genome and the PA14DING or PfluDING expression vector. The cell lysates were assayed for luciferase activity 48 hours post-transfection. PA14DING reduced HIV-1 production by 38% (p < 0.05) whereas PfluDING did not have a significant effect (Figure 4A).

The anti-HIV effect of PA14DING was verified by testing its effect on the production of the wild type HIV-1 virus. Microglial cells were transfected with the wild type pNL43 genome and PA14DING. The supernatant was assayed for the level of the viral capsid protein, p24, 48 hours post-transfection. PA14DING repressed viral production by ~43% (p < 0.05; Figure 4B). Taken all together our work suggests strongly that PA14DING is a potent inhibitor of HIV-1 replication.

### Identification of putative PA14DING pharmacophores

**Figure 5:**
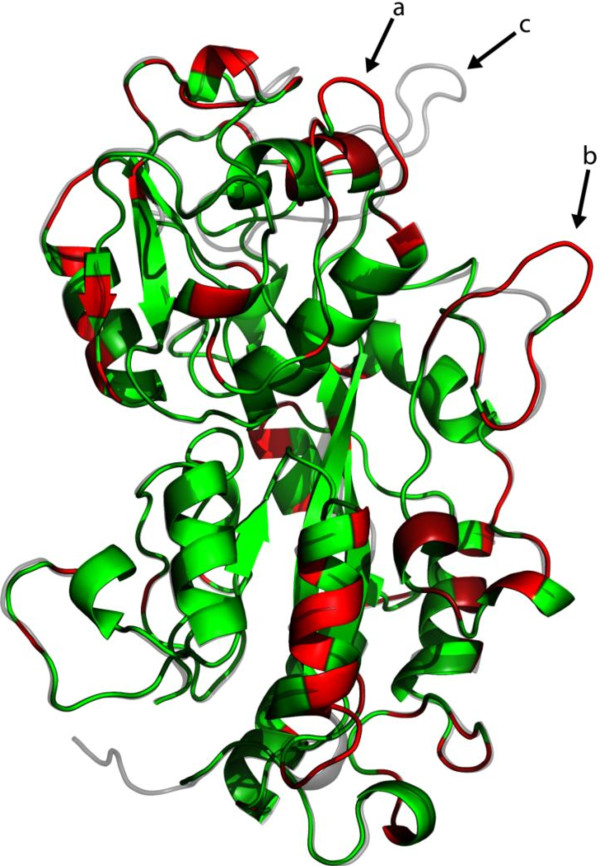
**The unique areas of PA14DING in comparison to PfluDING.** The LOMETS predicted model of PA14DING was overlaid with the crystal structure of PfluDING. The differences in sequence is between the two proteins were mapped red on the PA14DING model. Green: PA14DING model; Grey: PfluDING crystal structure. Differences in external loops are highlighted by arrows (**a**; residues 162–167) and (**b**; residues 269–275). Arrow **c** represents a loop present in PfluDING but absent in PA14.

PA14DING has a 74% sequence identity to PfluDING but the former was the only one to have anti-HIV activity. There were no significant differences in expression levels of the two proteins and this raises the possibility that peptide areas that are unique to PA14DING may be responsible for blocking HIV-1 transcription and replication. The predicted 3D structure of PA14DING was generated using the LOMETS algorithm and this was compared to the sub-angstrom crystal structure of PfluDING ([22]; Figure 5). The sequences that are unique to PA14DING were highlighted on its predicted structure. Three salient differences are seen in external loops of the upper globular domain, as the structures appear in Figure 5, and identified by arrows (a and b). These regions correspond to residues 162–167, and to 269–275, respectively, in the PA14DING amino acid sequence. In this region, the PfluDING molecule also has an additional loop (arrow c; residues 223–238) which is not present in PA14DING.

## Discussion

Previous studies with DING proteins from human disease contexts such as rheumatoid arthritis and cancer cachexia implicated DING proteins in having effects on NFκB-mediated transcription. It was not possible to clone a human DING gene for investigation due to a lack of full human DING DNA or mRNA sequences. Although the full amino acid sequence of the human phosphate-binding protein was known [19], attempts to express recombinant versions of the protein had been unsuccessful due to insolubility [3]. Whilst there was no previous compelling evidence of bacterial DING proteins affecting human transcription, the availability of full *Pseudomonas* DING genes from genome databases provided an opportunity to clone and directly assess the effect of DING proteins on NFκB mediated transcription. This study has shown that *Pseudomonas* DING proteins can significantly affect human transcription and be used as effective models for elucidating the function of eukaryotic DING proteins, including those isolated from human samples.

The direct stimulation of NFκB by DING proteins supports previous studies in which NFκB activation was directly observed, or implicated as the underlying mechanism causing observed phenotypic changes. In cancer cachexia, the stimulation of NFκB may explain the resulting activation of the ubiquitin-proteasome pathway and the consequent degradation of muscle and hence weight loss in cancer patients [9,10,27]. In rheumatoid arthritis, NFκB activation and IL-8 expression would explain the activation of synovial T-cells and the mitogenic stimulation of synovial fibroblasts [5,6]. *P. aeruginosa* PA14 is a human pathogen, and its ability to express and secrete a DING protein may elicit responses from host cells; many inflammatory responses are initiated by NFκB activation [28]. It is noteworthy that the stimulation of NFκB activity is unrelated to the phosphate-binding ability of PfluDING. We have not as yet created mutants of the phosphate-binding residues in PA14DING, so can make no definitive statement on the role of phosphate binding in anti-HIV-1 activity. However, as discussed below, the most prominent differences between the two proteins are distant from the phosphate-binding sites.

Both PA14DING and PfluDING stimulate NF-kB activity but PA14DING has a higher stimulatory effect and is the only one of the proteins to inhibit HIV-1 transcription and replication. The anti-HIV effect of the *P. aeruginosa* DING protein is consistent with accumulating evidence that eukaryotic DING proteins block HIV-1 transcription and replication [12-18,29]. It should be pointed out that, in the case of X-DING-CD4, the anti-HIV-1 effect is dependent upon the inhibition of NFκB action [15,16], whereas our results indicate that these two phenomena appear to be independent.

The two proteins are 74% identical in sequence and it is possible that regions where the two proteins share identical sequences are responsible for the stimulation of NFκB and a region that is unique to PA14DING is responsible for attenuating HIV-1 transcription and production. An overlay of the PfluDING crystal structure and PA14DING model highlights areas that are unique to PA14DING which may represent candidate areas that are responsible for the anti-HIV activity (Figure 5), and could form the basis for developing DING-based derivatives. The external loops which form the most obvious differences, are both in the domain which has been identified as responsible for the mitogenic activity of PfluDING towards human cells [21]. Although a truncated DING protein corresponding to this domain is very poorly soluble, the creation of full-length recombinant chimaeric PfluDING proteins, incorporating each of these sequences from PA14DING, should make it possible to identify the structural elements responsible for anti-HIV-1 activity, in combination with the use of site-directed mutants. Such information would provide the starting-point for the design of synthetic peptides which could mimic this activity.

The results from this study strongly suggest that PA14DING has a dual effect on HIV-1 transcription. Indeed, our work suggests that (i) PA14DING might activate the early cellular-dependant phase through NFκB activation and (ii) shows that PA14DING inhibits the Tat-dependent late phase [30]. Further investigation will be needed to show that PA14DING is involved in the activation of this early cell-dependent phase in microglial cells, which represent the main HIV reservoir in the central nervous system. Since it has been described previously that HIV-1 transcription and replication are activated through the NFκB pathway [31], it might be possible to design a peptide derivative in order to reactivate HIV-1 from these latent or quiescent infected microglial cells. It will therefore be very exciting to identify active PA14DING-derivatives involved in NFκB activation and HIV-1 transcription. A combination of both could be used in potential HIV-1 treatment strategies. The activation of HIV-1 transcription in the early phase could be used to re-activate latent viral reservoirs which then can be destroyed by cART [32]. In addition, as improving cART continues to be important, the inhibiting properties of PA14DING in the late phase of viral transcription could be used to complement current cART by further inhibiting HIV-1 replication and reducing viral load. A combination of these effects will also decrease the likelihood of the development of cART resistant HIV-1 strains [25]. The goal of this treatment strategy of reactivating latent viral reservoirs followed by their destruction, is to reduce the pool of latently-infected cells. Similarly, strategies based on early treatment intervention would aim to reduce the size of these latently-infected reservoirs. Developing a treatment employing both strategies may control HIV infection in patients, as observed in Elite Controllers, and therefore might lead to a functional cure.

## Conclusions

Both *Pseudomonas* DING proteins stimulate NFκB activity but PstS, a related phosphate-binding protein, does not. Mutation of the phosphate-binding site in a DING protein does not affect NF-κB stimulation. PA14DING is more effective as a stimulator of NFκB and is the only one of the proteins to inhibit HIV-1 transcription and production.

This study along with the X-DING-CD4, p38SJ, and HPBP studies provides evidence that DING-based derivatives could be effective anti-HIV agents. The recent identification of DING proteins originating from human samples that block HIV-1 replication and the identifica-tion of individuals with high DING mRNA expression correlating with HIV-1 resistance is evidence that DING proteins may effectively block HIV-1 replication *in vivo*[15-17]. The present study raises the possibility that *Pseudomonas* DING proteins could be further developed into anti-HIV molecules that would be easily administered and complement current combination therapy regimes.

## Methods

### Cloning

gDNA samples were extracted from overnight cultures of *P. fluorescens* SBW25 and *P. aeruginosa* PA14 using the PureLink™ Genomic DNA Mini Kit (Invitrogen) following the manufacturer’s guidelines. Primers were designed to clone the entire open reading frames of the PfluDING, PA14DING, Pflu PstS and PA14 PstS genes (excluding the predicted signal sequences). Primers contained a Kozak’s sequence (ACCATG) for mammalian expression, the stop codon was removed to allow the expression of a C-terminal V5 tag and were flanked with partial Gateway recombination site tags. A nested PCR approach was used to amplify the gene as previously described [33]. The final PCR product was inserted into the donor plasmid pDONR221 (Invitrogen) using the Gateway recombination system (Invitrogen) following the manufacturer’s protocols. Recombinant clones were screened for the presence of inserts by restriction enzyme digestion and verified by sequencing. A further recombination reaction and sequence verification was undertaken to insert the genes into pcDNA3.2/V5-DEST (Invitrogen) to produce DING and PstS mammalian expression vectors under the control of the CMV promoter (CMV-PfluDING, CMV-PA14DING, CMV-PfluPstS, CMV-PA14PstS).

### Site directed mutagenesis

Site-directed mutagenesis was undertaken using the overlap PCR method as previously described [21]. The resulting PCR products with appropriate mutations were cloned into pcDNA 3.2/V5-DEST vectors using the Gateway cloning system (Invitrogen).

### Cell culture

The two cell lines HEK 293T and microglial cells [34] were grown in Dulbecco’s modified Eagle medium (DMEM) supplemented with 10% fetal bovine serum and 100 ug/ml penicillin and streptomycin and glutamine.

### Reporter assays

The reporter plasmids for NFκB [35] and HIV-1 LTR [12] were kindly gifted by the authors of the respective papers. The IL-8 reporter plasmid and IkB super-repressor vectors were kindly gifted by Dr. Ashley Mansell.

HEK293T cells were transfected with reporter plasmids (expressing the firefly luciferase gene controlled by promoters), experimental plasmids and an internal control reporter encoding the Renilla luciferase gene (Promega) using the Lipofectamine 2000 reagent (Invitrogen) following the manufacturer’s protocol. Cells were assayed for luciferase activities 48 hours after transfection using the Dual Glo Luciferase Assay system (Promega) following the manufacturer’s protocol. Firefly luciferase activity was normalized against Renilla activity. The expression of overexpressed proteins was detected using SDS-PAGE and Western Blot analysis. Monoclonal anti-V5 antibodies (Invitrogen) were used to detect the expression of DING and PstS proteins co-expressed with C-terminal V5 tags and anti-β-actin antibodies (Sigma) were used to detect β-actin.

### HIV-1 production assay

Microglial cells were co-transfected with the HIV-1 pNL43 Δenv pseudovirus genome, in which the viral envelope gene had been replaced with a luciferase gene [36], with DING or PstS vectors using the calcium phosphate precipitation transfection method. Cells were assayed for luciferase activity 48 hours after transfection using the Luciferase Assay system (Promega) following the manufacturer’s protocol and the data were normalized by the total protein concentration.

For live HIV-1 production assays, microglial cells were transfected with the HIV-1 pNL43 genome using the calcium phosphate precipitation transfection method. The conditioned medium was collected 48 hours after transfection and levels of the viral capsid protein p24 were quantified using the INNOTEST HIV Antigen mAb ELISA kit following the manufacturer’s protocol.

In both cases we measured the luciferase activity or quantified the p24 capsid protein to determine the production of virus particles in a single round infection assay.

### 3D structure

The 3D model of PA14DING was produced using the LOMETS algorithm [37] and was viewed with Pymol. The PfluDING crystal structure [22] was obtained from the PDB.

### Statistics

The Student’s t-test was used to determine the significance of differences in data compared with the control. Data was considered significant with a p-value < 0.05.

## Competing interests

The authors declare that they have no competing interests.

## Authors’ contributions

KS and AS conceived the study, participated in its design and coordination and wrote the manuscript. AS undertook and participated in all experiments including cloning, site-directed mutagenesis, NFκB reporter assays in HEK 293T cells, HIV-1 transcription reporter assays in microglial cells, HIV-1 pNL43 Δenv pseudovirus reporter assays and HIV-1 pNL43 replication assays in microglial cells. VLD carried out HIV-1 pNL43 replication assays in microglial cells. OR and CS participated in the design of the study and drafting of the manuscript. All authors read and approved the final manuscript.

## References

[B1] Berna A Scott K Chabriere E Bernier F The DING family of proteins: ubiquitous in eukaryotes, but where are the genes? Bioessays 2009 10 570 580 19360767 10.1002/bies.200800174

[B2] Berna A Bernier F Chabriere E Perera T Scott K DING proteins; novel members of a prokaryotic phosphate-binding protein superfamily which extends into the eukaryotic kingdom Int J Biochem Cell Biol 2008 10 170 175 17368078 10.1016/j.biocel.2007.02.004

[B3] Berna A Bernier F Chabriere E Elias M Scott K Suh A For whom the bell tolls? DING proteins in health and disease Cell Mol Life Sci 2009 10 2205 2218 19290474 10.1007/s00018-009-0006-6 PMC11115607

[B4] Berna A Bernier F Scott K Stuhlmuller B Ring up the curtain on DING proteins FEBS Lett 2002 10 6 10 12135732 10.1016/s0014-5793(02)03053-3

[B5] Hain NA Stuhlmuller B Hahn GR Kalden JR Deutzmann R Burmester GR Biochemical characterization and microsequencing of a 205-kDa synovial protein stimulatory for T cells and reactive with rheumatoid factor containing sera J Immunol 1996 10 1773 1780 8759767

[B6] Adams L Davey S Scott K The DING protein: an autocrine growth-stimulatory protein related to the human synovial stimulatory protein Biochim Biophys Acta (BBA) Mol Basis Disease 2002 10 254 264 10.1016/s0925-4439(01)00104-1 11997077

[B7] Blass S Schumann F Hain NA Engel JM Stuhlmuller B Burmester GR p205 is a major target of autoreactive T cells in rheumatoid arthritis Arthritis Rheum 1999 10 971 980 10323453 10.1002/1529-0131(199905)42:5<971::AID-ANR16>3.0.CO;2-A

[B8] Tisdale MJ Cachexia in cancer patients Nat Rev Cancer 2002 10 862 871 12415256 10.1038/nrc927

[B9] Todorov P Cariuk P McDevitt T Coles B Fearon K Tisdale M Characterization of a cancer cachectic factor Nature 1996 10 739 742 8602222 10.1038/379739a0

[B10] Todorov PT Wyke SM Tisdale MJ Identification and characterization of a membrane receptor for proteolysis-inducing factor on skeletal muscle Cancer Res 2007 10 11419 11427 18056470 10.1158/0008-5472.CAN-07-2602

[B11] Watchorn TM Dowidar N Dejong CHC Waddell ID Garden J Ross JA The cachetic mediator proteolysis inducing factor activates NF-κB and STAT3 in human Kupffer cells and monocytes Int J Oncol 2005 10 1105 1111 16142329

[B12] Darbinian-Sarkissian N Darbinyan A Otte J Radhakrishnan S Sawaya BE Arzumanyan A Chipitsyna G Popov Y Rappaport J Amini S Khalili K p27SJ, a novel protein in St John's Wort, that suppresses expression of HIV-1 genome Gene Ther 2006 10 288 295 16251997 10.1038/sj.gt.3302649

[B13] Darbinian N Popov Y Khalili K Amini S Creation of a bi-directional protein transduction system for suppression of HIV-1 expression by p27SJ Antiviral Res 2008 10 136 141 18378326 10.1016/j.antiviral.2007.11.006 PMC2460567

[B14] Darbinian N Gomberg R Mullen L Garcia S White MK Khalili K Amini S Suppression of HIV-1 transcriptional elongation by a DING phosphatase J Cell Biochem 2011 10 225 232 21117063 10.1002/jcb.22915 PMC4503254

[B15] Lesner A Li Y Nitkiewicz J Li G Kartvelishvili A Kartvelishvili M Simm M A soluble factor secreted by an HIV-1-resistant cell line blocks transcription through inactivating the DNA-binding capacity of the NF-κB p65/p50 dimer J Immunol 2005 10 2548 2554 16081828 10.4049/jimmunol.175.4.2548

[B16] Lesner A Shilpi R Ivanova A Gawinowicz MA Lesniak J Nikolov D Simm M Identification of X-DING-CD4, a new member of human DING protein family that is secreted by HIV-1 resistant CD4(+) T cells and has anti-viral activity Biochem Biophys Res Commun 2009 10 284 289 19720052 10.1016/j.bbrc.2009.08.140 PMC2957897

[B17] Shilpi RY Sachdeva R Simm M Cellular resistance to HIV-1 infection in target cells coincides with a rapid induction of X-DING-CD4 mRNA: Indication of the unique host innate response to virus regulated through function of the X-DING-CD4 gene Innate Immun 2011 10.1177/1753425911426893 PMC3793846 22042911

[B18] Cherrier T Elias M Jeudy A Gotthard G Le Douce V Hallay H Masson P Janossy A Candolfi E Rohr O Human-Phosphate-Binding-Protein inhibits HIV-1 gene transcription and replication Virol J 2011 10 352 21762475 10.1186/1743-422X-8-352 PMC3157455

[B19] Morales R Berna A Carpentier P Contreras-Martel C Renault F Nicodeme M Chesne-Seck ML Bernier F Dupuy J Schaeffer C Serendipitous discovery and X-ray structure of a human phosphate binding apolipoprotein Structure 2006 10 601 609 16531243 10.1016/j.str.2005.12.012

[B20] Zhang XX Scott K Meffin R Rainey PB Genetic characterization of psp encoding the DING protein in *Pseudomonas fluorescens* SBW25 BMC Microbiol 2007 10 114 18088430 10.1186/1471-2180-7-114 PMC2225411

[B21] Ahn S Moniot S Elias M Chabriere E Kim D Scott K Structure-function relationships in a bacterial DING protein FEBS Lett 2007 10 3455 3460 17612529 10.1016/j.febslet.2007.06.050

[B22] Liebschner D Elias M Moniot S Fournier B Scott K Jelsch C Guillot B Lecomte C Chabriere E Elucidation of the phosphate binding mode of DING proteins revealed by subangstrom X-ray crystallography J Am Chem Soc 2009 10 7879 7886 19445459 10.1021/ja901900y

[B23] Scott K Wu L Functional properties of a recombinant bacterial DING protein: comparison with a homologous human protein Biochim Biophys Acta 2005 10 234 244 15950753 10.1016/j.bbamcr.2005.02.003

[B24] Domingo P Vidal F Combination antiretroviral therapy Expert Opin Pharmacother 2011 10 995 998 21434758 10.1517/14656566.2011.567001

[B25] Le Douce V Janossy A Hallay H Ali S Riclet R Rohr O Schwartz C Achieving a cure for HIV infection: do we have reasons to be optimistic? J Antimicrob Chemother 2012 10 1063 1074 22294645 10.1093/jac/dkr599 PMC3324423

[B26] Shafer RW Schapiro JM HIV-1 drug resistance mutations: An updated framework for the second decade of HAART AIDS Rev 2008 10 67 84 18615118 PMC2547476

[B27] Whitehouse AS Tisdale MJ Increased expression of the ubiquitin-proteasome pathway in murine myotubes by proteolysis-inducing factor (PIF) is associated with activation of the transcription factor NF-κB Br J Cancer 2003 10 1116 1122 12966435 10.1038/sj.bjc.6601132 PMC2376944

[B28] Tripathi P Aggarwal A NF-κB transcription factor: a key player in the generation of immune response Curr Sci 2006 10 519 531

[B29] Darbinian N Czernik M Darbinyan A Elias M Chabriere E Bonasu S Khalili K Amini S Evidence for phosphatase activity of p27SJ and its impact on the cell cycle J Cell Biochem 2009 10 400 407 19343785 10.1002/jcb.22135 PMC2769080

[B30] Rohr O Marban C Aunis D Schaeffer E Regulation of HIV-1 gene transcription: from lymphocytes to microglial cells J Leukoc Biol 2003 10 736 749 12960235 10.1189/jlb.0403180

[B31] Janabi N di Stefano M Wallon C Hery C Chiodi F Tardieu M Induction of human immunodeficiency virus type 1 replication in human glial cells after proinflammatory cytokines stimulation: effect of IFNγ, IL1β and TNFα on differentiation and chemokine production in glial cells Glia 1998 10 304 315 9671961

[B32] Le Douce V Herbein G Rohr O Schwartz C Molecular mechanisms of HIV-1 persistence in the monocyte-macrophage lineage Retrovirology 2010 10 32 20380694 10.1186/1742-4690-7-32 PMC2873506

[B33] Moreland N Ashton R Baker HM Ivanovic I Patterson S Arcus VL Baker EN Lott JS A flexible and economical medium-throughput strategy for protein production and crystallization Acta Crystallogr D Biol Crystallogr 2005 10 1378 1385 16204890 10.1107/S0907444905023590

[B34] Janabi N Peudenier S Heron B Ng KH Tardieu M Establishment of human microglial cell lines after transfection of primary cultures of embryonic microglial cells with the SV40 large T antigen Neurosci Lett 1995 10 105 108 7478261 10.1016/0304-3940(94)11792-h

[B35] Kopp E Ghosh S Inhibition of NF-kappa B by sodium salicylate and aspirin Science 1994 10 956 959 8052854 10.1126/science.8052854

[B36] Le Douce V Colin L Redel L Cherrier T Herbein G Aunis D Rohr O Van Lint C Schwartz C LSD1 cooperates with CTIP2 to promote HIV-1 transcriptional silencing Nucleic Acids Res 2012 10 1904 1915 22067449 10.1093/nar/gkr857 PMC3300010

[B37] Wu S Zhang Y LOMETS: a local meta-threading-server for protein structure prediction Nucleic Acids Res 2007 10 3375 3382 17478507 10.1093/nar/gkm251 PMC1904280

